# Gene Therapy for Cardiac Arrhythmias: Mechanisms, Modalities and Therapeutic Applications

**DOI:** 10.3390/medsci13030102

**Published:** 2025-07-30

**Authors:** Paschalis Karakasis, Panagiotis Theofilis, Panayotis K. Vlachakis, Nikias Milaras, Kallirhoe Kalinderi, Dimitrios Patoulias, Antonios P. Antoniadis, Nikolaos Fragakis

**Affiliations:** 1 Second Department of Cardiology, Hippokration General Hospital, Aristotle University of Thessaloniki, 54642 Thessaloniki, Greece; aantoniadis@gmail.com (A.P.A.); fragakis.nikos@googlemail.com (N.F.); 2 First Cardiology Department, School of Medicine, Hippokration General Hospital, National and Kapodistrian University of Athens, 15772 Athens, Greece; panos.theofilis@hotmail.com (P.T.); vlachakispanag@gmail.com (P.K.V.); nikiasmilaras@gmail.com (N.M.); 3 Department of Biomedical Sciences, School of Health Sciences, International Hellenic University, 5400 Thessaloniki, Greece; roey111@hotmail.com; 4 Second Propedeutic Department of Internal Medicine, Faculty of Medicine, School of Health Sciences Aristotle, University of Thessaloniki, 54124 Thessaloniki, Greece; dipatoulias@gmail.com

**Keywords:** cardiac arrhythmias, gene therapy, ion channelopathies, genetic modulation, atrial fibrillation, ventricular tachycardia, viral vectors, genome editing

## Abstract

Cardiac arrhythmias remain a major source of morbidity and mortality, often stemming from molecular and structural abnormalities that are insufficiently addressed by current pharmacologic and interventional therapies. Gene therapy has emerged as a transformative approach, offering precise and durable interventions that directly target the arrhythmogenic substrate. Across the spectrum of inherited and acquired arrhythmias—including long QT syndrome, Brugada syndrome, catecholaminergic polymorphic ventricular tachycardia, atrial fibrillation, and post-infarction ventricular tachycardia—gene-based strategies such as allele-specific silencing, gene replacement, CRISPR-mediated editing, and suppression-and-replacement constructs are showing growing translational potential. Advances in delivery platforms, including cardiotropic viral vectors, lipid nanoparticle-encapsulated mRNA, and non-viral reprogramming tools, have further enhanced the specificity and safety of these approaches. Additionally, innovative applications such as biological pacemaker development and mutation-agnostic therapies underscore the versatility of genetic modulation. Nonetheless, significant challenges remain, including vector tropism, immune responses, payload limitations, and the translational gap between preclinical models and human electrophysiology. Integration of patient-derived cardiomyocytes, computational simulations, and large-animal studies is expected to accelerate clinical translation. This review provides a comprehensive synthesis of the mechanistic rationale, therapeutic strategies, delivery platforms, and translational frontiers of gene therapy for cardiac arrhythmias.

## 1. Introduction

Cardiac arrhythmias remain a major global health challenge, contributing substantially to morbidity, mortality, and healthcare burden across a wide spectrum of acquired and inherited cardiovascular conditions [1,2,3,4]. While advances in pharmacologic agents and device-based therapies have improved arrhythmia management, current treatment paradigms often remain palliative, targeting downstream electrical manifestations without addressing the underlying molecular substrate [5,6]. This therapeutic gap is particularly evident in genetically mediated arrhythmias—such as long QT syndrome, Brugada syndrome, catecholaminergic polymorphic ventricular tachycardia (CPVT), and short QT syndrome—as well as in acquired forms where maladaptive ion channel remodeling and fibrotic signaling underlie arrhythmogenic risk [7,8,9].

Recent decades have witnessed remarkable progress in the identification of genetic and molecular mechanisms contributing to arrhythmogenesis [10,11,12,13]. Advances in high-throughput sequencing and functional genomics have elucidated a complex landscape of pathogenic variants and signaling pathways involved in ion homeostasis, membrane excitability, calcium cycling, and intercellular conduction [14]. These insights have catalyzed a paradigm shift toward mechanism-guided, gene-targeted therapies that aim not only to suppress arrhythmias, but to modify or correct the arrhythmogenic substrate at its source [15,16].

Alongside these developments, pharmacogenetics has emerged as a useful tool for personalizing arrhythmia therapy [17]. Variants in ion channel genes, such as SCN5A, KCNQ1, and KCNA5, as well as in drug-metabolizing enzymes like CYP2D6 and CYP3A5, have been shown to influence both arrhythmia risk and treatment response [18,19]. For example, SCN5A mutations may change how sodium channel blockers work, while KCNQ1 polymorphisms can affect repolarization reserve and sensitivity to Class III agents [20]. Additionally, genome-wide association studies have connected several common variants to atrial fibrillation risk, ablation outcomes, and negative drug responses [21,22]. Adding pharmacogenetic insights into clinical practice may help refine treatment choices and improve safety profiles, providing a complementary strategy to gene therapy in the larger context of precision electrophysiology.

**Figure 1:**
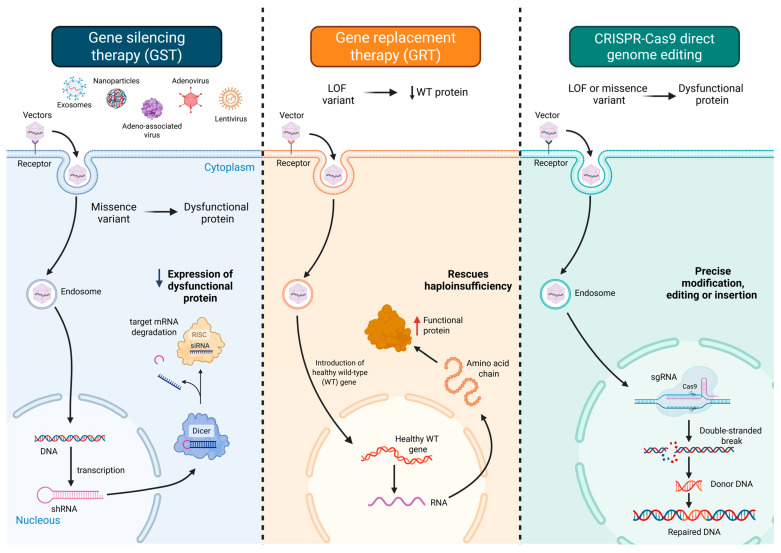
Overview of gene therapy strategies for managing cardiac arrhythmias. Gene silencing therapy (left panel) attenuates the expression of pathogenic alleles via antisense oligonucleotides (ASOs) or RNA interference modalities such as small interfering RNAs (siRNAs), thereby counteracting deleterious gain-of-function effects. Gene replacement therapy (middle panel) delivers exogenous wild-type cDNA to restore functional protein levels, effectively addressing haploinsufficiency or complete loss-of-function in monogenic cardiac diseases. Direct genome editing (right panel) enables precise modification of the host genome through CRISPR-Cas9-mediated double-stranded DNA cleavage, base editing for targeted nucleotide substitutions, or prime editing for programmable insertions and deletions without inducing DNA breaks. Additional therapeutic modalities include pathway modulation, which aims to restore homeostasis by targeting key nodes within disease-relevant signaling cascades—often bypassing direct gene correction—and suppression-and-replacement (SupRep) approaches, which concurrently silence both wild-type and mutant alleles using short hairpin RNA, while reintroducing a knockdown-resistant transgene to reconstitute protein function. Optimization strategies across all modalities involve tailored vector selection (e.g., adeno-associated viruses and lipid nanoparticles), cardiac-specific promoters, and advanced delivery systems to enhance myocardial targeting and minimize off-target effects. Abbreviations: AAV, adeno-associated virus; Cas9, CRISPR-associated protein 9; DNA, deoxyribonucleic acid; GST, gene silencing therapy; GRT, gene replacement therapy; LOF, loss-of-function; mRNA, messenger RNA; RISC, RNA-induced silencing complex; RNA, ribonucleic acid; shRNA, short hairpin RNA; siRNA, small interfering RNA; sgRNA, single guide RNA; WT, wild type.

Emerging gene-based therapies offer a diverse and increasingly refined toolkit to modulate arrhythmia-related molecular circuits. These strategies include gene silencing therapy (GST), which attenuates the expression of pathogenic alleles via antisense oligonucleotides (ASOs) [23,24], RNA interference (RNAi) [25,26], or CRISPR interference (CRISPRi) [27]; gene replacement therapy (GRT), wherein exogenous delivery of wild-type cDNA restores physiological protein function [28]; and direct genome editing, which enables permanent correction of disease-causing mutations through CRISPR-Cas9 or base/prime editing platforms [29] (Figure 1). In addition, pathway modulation therapies target downstream or compensatory signaling cascades to ameliorate functional deficits [30], while suppression-and-replacement (SupRep) therapy offers a hybrid strategy by simultaneously silencing endogenous gene expression and introducing a knockdown-resistant wild-type transgene [31].

This review explores gene modulation strategies for inherited and acquired cardiac arrhythmias, focusing on their mechanistic basis and delivery platforms while emphasizing their translational potential from preclinical models to future clinical application.

## 2. Genetic Therapeutic Strategies in the Management of Cardiac Arrhythmias

### 2.1. Molecular Modulation of Electrophysiological Substrates in Atrial Fibrillation

Atrial fibrillation (AF) represents the most prevalent sustained atrial arrhythmia and is associated with an elevated risk of thromboembolic events, heart failure, and all-cause mortality [3,32,33,34,35,36,37,38]. Conventional rhythm control strategies—primarily relying on antiarrhythmic pharmacotherapy and catheter ablation—often demonstrate suboptimal efficacy, with long-term administration constrained by significant adverse effects [39]. These limitations underscore the pressing need for novel, mechanism-targeted interventions such as gene therapy. The complex pathophysiology of AF is underpinned by focal ectopic activity and re-entrant circuits, both of which are potentiated by a substrate of electrical and structural remodeling [40,41,42]. Concomitant alterations in autonomic regulation and intracellular calcium homeostasis further contribute to the arrhythmogenic milieu, providing potential molecular targets for therapeutic modulation [43].

Electrical remodeling in AF is characterized by a progressive shortening of atrial action potential duration (APD), primarily driven by a reduction in the L-type calcium current (I_Ca,L_), an upregulation of the inward rectifier potassium current (I_K1_), and enhanced activity of constitutively active acetylcholine-gated potassium currents (I_KACh_) [44,45,46,47]. These alterations facilitate re-entry and increase the susceptibility to arrhythmogenesis [48]. Gene therapy has emerged as a promising strategy to counteract such electrophysiological disturbances by targeting specific ion channels to restore a more physiological APD [49].

One of the most extensively investigated targets is the rapid delayed rectifier potassium current (I_Kr_), mediated by the K_v11.1_ channel, whose α-subunit is encoded by KCNH2. In preclinical porcine models of AF, adenoviral delivery of a dominant-negative KCNH2 variant led to significant prolongation of atrial APD, effectively suppressing AF inducibility and facilitating spontaneous reversion to sinus rhythm [50,51]. Parallel investigations have explored other pivotal ion channels implicated in atrial repolarization abnormalities, including the L-type calcium channel [52] and the voltage-gated potassium channel K_v1.5_ [53], uncovering additional avenues for therapeutic modulation.

Beyond ion channel regulation, gene therapy has been utilized to restore impaired intercellular electrical coupling. Gap junction dysfunction, particularly involving connexin 40 (Cx40) and connexin 43 (Cx43), plays a critical role in the maintenance of AF [54,55]. Epicardial gene transfer via adenoviral vectors—delivered either by injection or electroporation—has been shown to reconstitute connexin expression, improve atrial conduction velocity, and promote rhythm stabilization in large-animal models [54,55] (Table 1).

Furthermore, autonomic remodeling, especially heightened parasympathetic tone, has been implicated in AF pathogenesis [64]. Aberrant G-protein-mediated signaling involving Gα_iβγ subunits contributes to vagally induced AF. Peptide-based inhibition of these G-protein complexes in canine models has resulted in marked attenuation of AF susceptibility under vagal stimulation [58], offering an additional molecular target for anti-arrhythmic gene therapy.

While gene therapy strategies directed at modulating electrical remodeling have demonstrated substantial efficacy in attenuating AF in preclinical models, emerging efforts are also focused on the molecular underpinnings of structural remodeling that sustain the arrhythmic substrate. Among these, atrial fibrosis represents a pivotal contributor to conduction heterogeneity and AF maintenance. Transforming growth factor-β (TGF-β), a central mediator of fibrotic remodeling, has been successfully targeted using gene replacement therapy to induce atrial overexpression of a dominant-negative TGF-β variant in canine models [56]. This intervention markedly attenuated profibrotic signaling, reduced interstitial fibrosis, and improved conduction uniformity [56].

In parallel, gene silencing approaches aimed at mitigating myocyte apoptosis—another key driver of adverse atrial remodeling—have shown promise. Specifically, small interfering RNA (siRNA)-mediated inhibition of caspase-3, a principal executioner of apoptosis, has been employed in porcine models to suppress apoptotic pathways [57]. This strategy resulted in reduced cardiomyocyte loss and delayed progression to sustained AF [57]. Collectively, these approaches highlight the expanding therapeutic landscape of gene therapy in AF, extending beyond ion channel modulation to encompass key structural and cellular processes integral to arrhythmia perpetuation [39].

### 2.2. Ventricular Arrhythmias

Ventricular arrhythmias, encompassing ventricular tachycardia (VT) and ventricular fibrillation (VF), represent critical clinical entities with a high risk of sudden cardiac death, often necessitating immediate and sustained therapeutic intervention [38,65]. While pharmacologic agents such as β-adrenergic blockers and amiodarone remain foundational in arrhythmia management, their efficacy is frequently limited, and long-term use is burdened by substantial adverse effect profiles [66,67]. Implantable cardioverter-defibrillators (ICDs), though highly effective in terminating life-threatening arrhythmias and improving survival, are associated with significant device-related complications, including inappropriate shocks, lead dysfunction, and infection risk [68].

Given these limitations, gene therapy has emerged as a potential precision-based strategy to address the underlying molecular substrates of both acquired and inherited ventricular arrhythmias. In this section, we critically examine the current landscape of gene-based interventions for ventricular arrhythmogenesis, highlighting key preclinical advances and emerging therapeutic targets.

#### 2.2.1. Acquired Ventricular Arrhythmias

Acquired ventricular arrhythmias commonly result from pathological alterations in myocardial electrophysiology secondary to structural heart disease or systemic insults [69,70,71,72,73,74,75]. Among the most prominent etiologies is coronary artery disease, wherein myocardial ischemia or post-infarction fibrosis establishes an arrhythmogenic substrate that predisposes to malignant ventricular rhythms [70,75]. In particular, delayed conduction within infarct border zones promotes re-entrant circuits, culminating in sustained ventricular tachycardia [76].

Gene therapy offers a novel approach to interrupt these arrhythmogenic pathways by directly modifying molecular and electrophysiological determinants of conduction. One notable strategy involves the use of adenoviral vectors to overexpress the skeletal muscle sodium channel (SkM1, encoded by SCN4A) in the infarcted myocardium. This intervention has been shown to restore excitability and enhance conduction velocity in the depolarized epicardial border zones, where the native cardiac sodium channel (SCN5A) is often inactivated following myocardial infarction, thereby suppressing the initiation of re-entrant ventricular tachycardia [61]. In parallel, gene-based delivery of non-coding RNAs has been investigated to attenuate ischemia-induced remodeling, stimulate angiogenesis, and promote myocardial regeneration—further contributing to arrhythmia prevention [77,78,79,80].

#### 2.2.2. Inherited Ventricular Arrhythmias

**Figure 2:**
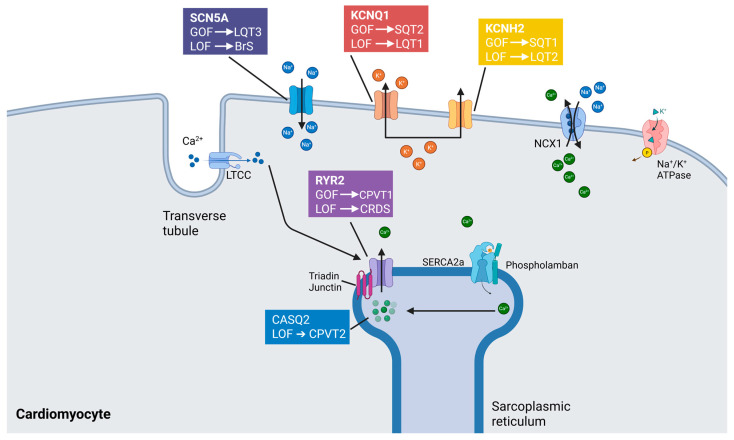
Mechanistic basis of selected inherited cardiac arrhythmia syndromes. Inherited cardiac arrhythmias arise from pathogenic variants in key genes encoding proteins involved in cardiac excitability, conduction, and calcium handling. These genetic alterations—typically manifesting as either loss-of-function (LOF) or gain-of-function (GOF) mutations—disrupt the finely tuned balance of ion fluxes and intracellular signaling, culminating in distinct electrophysiologic phenotypes. Among the principal clinical entities are Brugada syndrome (BrS), catecholaminergic polymorphic ventricular tachycardia (CPVT), calcium-release deficiency syndrome (CRDS), long QT syndrome (LQTS), and short QT syndrome (SQTS). Each disorder is linked to specific molecular derangements: for example, L-type calcium channels (LTCCs), sodium-calcium exchanger 1 (NCX1), and sarcoplasmic/endoplasmic reticulum calcium ATPase 2a (SERCA2a) represent pivotal components of calcium cycling pathways whose dysfunction contributes to arrhythmogenesis. Abnormal phosphorylation states and impaired protein-protein interactions within these signaling cascades further modulate arrhythmic risk. The resultant electrical instability underscores the importance of precise ion channel regulation and excitation-contraction coupling integrity in maintaining cardiac rhythm.

Inherited cardiac channelopathies represent a heterogeneous group of arrhythmogenic disorders, encompassing Brugada syndrome, catecholaminergic polymorphic ventricular tachycardia (CPVT), long QT syndrome (LQTS), and short QT syndrome [38,81] (Figure 2). These conditions are typically caused by pathogenic variants in genes encoding cardiac ion channels or associated regulatory proteins, resulting in abnormal myocardial excitability and conduction [82]. Current management strategies remain largely palliative, aiming to mitigate arrhythmic risk rather than directly correcting the underlying molecular defect. Conventional therapies—such as β-adrenergic blockers, implantable cardioverter-defibrillators (ICDs), and surgical sympathetic denervation—are frequently associated with substantial adverse effects that compromise long-term adherence and patient quality of life [83]. β-blockers, while effective in reducing arrhythmic events, are often poorly tolerated due to fatigue, depressive symptoms, and exercise intolerance [84,85]. Cardiac sympathetic denervation, though beneficial in selected cases, may lead to persistent neuropathic pain [86]. Likewise, ICD implantation is associated with a risk of device-related complications, including inappropriate shocks, infections, and psychological distress, such as post-traumatic stress disorder.

These limitations underscore the need for disease-modifying therapies, with gene therapy emerging as a particularly promising strategy. By directly targeting the arrhythmogenic substrate, gene therapy holds the potential to provide a durable solution, especially for patients with severe genotypes—such as malignant SCN5A variants (e.g., R1623Q)—or those unable to tolerate standard interventions [87]. Moreover, gene therapy may overcome the challenge of treatment adherence by offering a long-term, potentially single-dose therapeutic alternative capable of restoring physiological electrophysiologic function.

CPVT is an inherited arrhythmia syndrome characterized by adrenergically induced ventricular tachyarrhythmias and a heightened risk of sudden cardiac death in young individuals [88]. CPVT type 1 (CPVT1), the most prevalent form, is caused by autosomal dominant mutations in RYR2, which encodes the cardiac ryanodine receptor 2—an essential calcium-release channel located on the sarcoplasmic reticulum [89]. To date, over 150 distinct pathogenic RYR2 variants have been identified, all of which promote aberrant diastolic calcium leakage, thereby triggering delayed afterdepolarizations and fatal ventricular arrhythmias. Conversely, CPVT type 2 (CPVT2) follows an autosomal recessive inheritance pattern and results from loss-of-function mutations in CASQ2, which encodes calsequestrin-2, a critical calcium-buffering protein within the sarcoplasmic reticulum [90]. Disruption of this buffering capacity similarly leads to intracellular calcium instability and heightened arrhythmogenicity. In response to the limited efficacy of current pharmacologic therapies, a range of gene-based interventions has been explored in preclinical models of CPVT [91].

Researchers demonstrated the therapeutic efficacy of adeno-associated virus serotype 9 (AAV9)-mediated gene replacement therapy in a murine model of CPVT2 [92] (Table 2). By delivering wild-type CASQ2 to Casq2-knockout mice, they effectively abolished adrenergically induced ventricular arrhythmias, thereby providing proof-of-concept for gene therapy in autosomal recessive CPVT. A similar approach was subsequently applied to a CASQ2^R33Q^ knock-in model, yielding sustained anti-arrhythmic protection for up to one year following a single vector administration—highlighting the durability and translational potential of this intervention [93]. Importantly, the therapeutic applicability of CASQ2 overexpression extends beyond CPVT2. In a separate study, augmentation of CASQ2 expression in a model of RYR2-mediated CPVT1 significantly mitigated arrhythmia susceptibility [94]. This strategy exemplifies the concept of targeting nodal points within shared molecular pathways—rather than the defective gene itself—to achieve phenotypic correction. Such a pathway-directed approach is particularly valuable in the context of RYR2-related CPVT1, where traditional gene replacement therapy remains technically unfeasible due to the exceptional size of the RYR2 coding sequence, which spans nearly 15,000 nucleotides and exceeds the packaging capacity of current viral vectors.

Beyond primary channelopathies like CPVT, issues with calcium handling are also key to the arrhythmogenic basis of various cardiomyopathies, including hypertrophic, dilated, and arrhythmogenic right ventricular cardiomyopathy [95,96,97]. In this context, gene therapies that target RyR2 stabilization, CaMKII inhibition, or calmodulin modulation—originally developed for inherited ventricular arrhythmias—could also help reduce ventricular arrhythmias caused by structural heart disease [95,96,98]. For example, excessive RyR2-mediated calcium leak and CaMKII overactivation are involved in both ventricular ectopy and mechanical dysfunction in heart failure [98]. Therefore, strategies for gene modulation based on these mechanisms may provide a double benefit: reducing arrhythmogenic triggers while stabilizing the cardiomyopathic substrate [99,100]. This therapeutic overlap highlights the potential of shared targets that focus on the electromechanical interface in cardiomyopathy-related ventricular arrhythmias.

Gene-silencing strategies have been employed to selectively suppress pathogenic Ryr2 alleles in murine models of CPVT1, yielding significant suppression of ventricular arrhythmias. Notably, this approach not only ameliorated the arrhythmic phenotype but also restored ultrastructural integrity within the junctional sarcoplasmic reticulum and transverse tubular network, which are frequently disrupted in CPVT [101]. In parallel, genome-editing techniques have shown promise in correcting specific RYR2 mutations in vivo. Using CRISPR-Cas9, researchers successfully targeted the RYR2^R176Q^ variant in heterozygous mice, resulting in normalization of calcium handling and suppression of arrhythmogenesis [102]. Despite these successes, both allele-specific silencing and gene editing approaches currently face translational barriers, including the necessity for developing distinct, variant-specific therapeutic constructs—an approach that may be impractical given the vast mutational heterogeneity of RYR2.

To overcome these limitations, pathway-targeted interventions have emerged as a mutation-independent therapeutic alternative. One such strategy involved AAV9-mediated myocardial delivery of a peptide inhibitor of calcium/calmodulin-dependent protein kinase II (CaMKII) in RYR2^R176Q^ mice, which successfully suppressed spontaneous calcium release and ventricular arrhythmias [103]. In another study, AAV9 was used to deliver a modified form of calmodulin—engineered to enhance negative regulation of RYR2 channel opening—in CASQ2^R33Q^ mice [104]. This intervention increased ryanodine receptor refractoriness during repolarization, effectively preventing arrhythmic events [104].

LQTS is predominantly driven by loss-of-function, dominant-negative mutations in the potassium channel genes KCNQ1 (LQTS type 1) and KCNH2 (LQTS type 2), both of which play essential roles in cardiac repolarization [105,106,107]. Although allele-specific RNA interference (RNAi) strategies have been employed to selectively target pathogenic variants in KCNQ1 and KCNH2, their broader clinical applicability is hindered by the extreme mutational heterogeneity of these genes—each harboring hundreds of rare, disease-causing variants without a single predominant mutation. As a result, the field is shifting toward the development of variant-agnostic therapeutic approaches.

To this end, the SupRep (suppression-replacement) gene therapy platform has been engineered to address the limitations of allele-specific targeting by simultaneously silencing endogenous mutant transcripts and reintroducing a functional, suppression-resistant cDNA. In both human induced pluripotent stem cell-derived cardiomyocytes (hiPSC-CMs) [108] and a transgenic rabbit model of LQTS type 1 [109], SupRep therapy successfully restored physiological repolarization, normalizing QT intervals and action potential durations to wild-type levels [110]. The approach has also been extended to hiPSC-CM models of LQTS type 2 (KCNH2 mutations), short QT syndrome type 1 [31], and calmodulin-associated LQTS [111], demonstrating its versatility across multiple genotypes and channelopathies. 

**Table 2:** Key Studies Investigating Gene Therapy Strategies for Inherited Cardiac Arrhythmias.

Author (Year)	Type of Arrhythmia	Type of Study	Vector	Form of Gene Therapy	Main Findings
Yu et al. (2022) [112]	Brugada syndrome (BrS), ventricular tachyarrhythmias (VTs), sinus node dysfunction (SND), cardiac conduction disease (CCD)	Preclinical in vivo (murine KI models and hiPSC-derived cardiomyocytes	AAV9	AAV9-mediated overexpression of MOG1 (20 kDa protein chaperone for NaV1.5)	AAV9-MOG1 significantly increased Na_V1.5_ surface expression and peak INa density (e.g., from −7.68 ± 0.52 to −16.02 ± 1.27 pA/pF in Scn5aG1746R/+ mice; *p* < 0.001). It reversed abnormal ECG features (abolished J waves, sinus arrest, and VT), restored shortened APD (APD100 and APD90), and eliminated late phase 3 EADs. It downregulated Kcnd3 and Cacna1c expression. In SCN5A-D1275N KI mice, it rescued contractile dysfunction (EF and FS restored), reduced sinus pauses and heart block incidence, and increased stroke volume and cardiac output (SV: 24.55 μL vs. 31.42 μL; *p* = 0.0133). Similar rescue of INa density was seen in hiCMs (e.g., −11.33 to −15.81 pA/pF; *p* = 0.022).
Santiago Castillo et al. (2023) [94]	Catecholaminergic polymorphic ventricular tachycardia type 1 (CPVT1)	In silico model + in vivo (RyR2 R4496C/+ KI mice)	AAV (adeno-associated viral vector)	AAV-mediated overexpression of CASQ2 (cardiac calsequestrin)	In silico: Arrhythmia suppression achieved with 1.4-fold CASQ2 overexpression for Class 1 RyR2 mutations and 1.8-fold for Class 2. In vivo: CASQ2 gene therapy completely prevented arrhythmias upon caffeine/epinephrine challenge (0/12 treated vs. 17/32 untreated mice; *p* = 0.0012), showing potent antiarrhythmic efficacy and validating CASQ2 overexpression as a novel therapeutic approach in CPVT1.
Bongianino et al. (2017) [101]	Catecholaminergic polymorphic ventricular tachycardia (CPVT1)	In vivo (CASQ2^R33Q/R33Q^ knock-in mouse model)	Liposome-mediated delivery (Invivofectamine)	Allele-specific silencing of mutant CASQ2 mRNA via siRNA	Systemic delivery of siCASQ2-R33Q (5 mg/kg) significantly reduced mutant CASQ2 mRNA and protein expression by ~60%, without affecting WT CASQ2. After 3 injections (every 2 weeks), treated mice showed normalization of calcium release, reduced spontaneous Ca^2+^ waves, and prevention of arrhythmias induced by epinephrine/caffeine challenge (0/9 treated vs. 8/12 untreated mice; *p* = 0.003). Electron microscopy revealed restored junctional SR architecture. No toxicity or off-target effects observed.
Pan et al. (2023) [102]	Catecholaminergic polymorphic ventricular tachycardia (CPVT1)	In vivo (RyR2^R4496C^/+ knock-in mouse model)	AAV9	CRISPR-based gene editing using AAV9-Staphylococcus aureus Cas9 (SaCas9) and guide RNA	AAV9-SaCas9-mediated editing targeted mutant RYR2^R4496C^ in the heart, achieving ~41% editing efficiency in cardiomyocytes. Treated mice exhibited significant reduction in ventricular arrhythmias during epinephrine/caffeine challenge (0/7 treated vs. 7/8 control; *p* = 0.0014). Restored Ca^2+^ handling was observed with decreased spontaneous Ca^2+^ waves and normalized Ca^2+^ transients. No significant off-target editing detected in vivo, and no adverse effects noted. This study offers first in vivo demonstration of safe, effective CRISPR-mediated RYR2 repair in a CPVT1 model.
Bezzerides et al. (2019) [103]	Catecholaminergic polymorphic ventricular tachycardia (CPVT1)	In vivo (CASQ2 knockout mice) + isolated cardiomyocytes	AAV9	AAV9-mediated delivery of CaMKII-inhibitory peptide (AIP)	AAV9-AIP significantly reduced ventricular arrhythmias in CASQ2−/− mice during epinephrine/challenge (event rate 0.67 vs. 2.17 events/min in controls; *p* < 0.01). Optical mapping showed normalization of Ca^2+^ transients and suppression of diastolic Ca^2+^ waves. Treated cardiomyocytes displayed reduced spontaneous Ca^2+^ oscillations (27% vs. 79% in untreated; *p* < 0.01). AIP therapy was well tolerated with no off-target contractility or structural effects, demonstrating efficacy of targeted CaMKII inhibition in genetic arrhythmia.
Denegri et al. (2012) [92]	Catecholaminergic polymorphic ventricular tachycardia (CPVT, CASQ2-related)	In vivo (CASQ2 knockout mice)	AAV9	AAV9-mediated delivery of wild-type CASQ2 gene	Viral delivery of CASQ2 infected ~50% of myocytes, restored CASQ2, triadin (TrD), and junction (JnC) levels to ~80–90% of wild-type levels. This led to reversal of jSR structural abnormalities, including jSR width normalization (from 37 ± 1.2 nm in KO to 21 ± 0.3 nm in treated; *p* < 0.001), reduced triggered activity during β-adrenergic stimulation (from 70% to 5% of myocytes; *p* < 0.001), and suppression of in vivo ventricular tachycardia (from 15/15 to 1/10 mice after epinephrine; *p* < 0.001). No histological toxicity observed. Demonstrated durable structural and functional rescue.
Denegri et al. (2014) [93]	Catecholaminergic polymorphic ventricular tachycardia (CPVT1, CASQ2-R33Q model)	In vivo (CASQ2^R33Q/R33Q^ knock-in mice)	AAV9	AAV9-mediated delivery of wild-type CASQ2 gene	A single intravenous AAV9-CASQ2 injection (3.5 × 10^13^ vg/kg) restored CASQ2 protein expression to ~60% of WT levels, reversed RyR2 mislocalization, and normalized jSR ultrastructure. Treated mice showed >85% reduction in arrhythmia incidence during exercise + epinephrine challenge (from 92% to 8%; *p* < 0.001), and >90% reduction in premature ventricular contractions. Intracellular Ca^2+^ cycling was restored with normalization of Ca^2+^ transients and reduced spontaneous waves. Effects were sustained for at least 3 months with no observed toxicity, confirming long-term efficacy and safety.
Bains et al. (2024) [110]	Congenital Long QT Syndrome Type 1 (LQT1)	Preclinical (animal model—transgenic rabbit)	AAV9	KCNQ1 suppression-and-replacement (SupRep)	The therapy combined shRNA-mediated suppression of endogenous KCNQ1 and replacement with shRNA-immune KCNQ1 cDNA. In vivo administration (1E10 vg/kg, intra-aortic root injection) led to significant QT index (QTi) shortening in LQT1 rabbits (from 122 ± 3% to 110 ± 4%, *p* = 0.03), normalizing levels close to wild type (WT: 105 ± 2%). APD90 in ventricular cardiomyocytes decreased from 525 ± 15 ms (untreated LQT1) to 394 ± 15 ms (treated), approaching WT values (417 ± 14 ms). Under β-adrenergic stimulation, SupRep-treated animals exhibited normal physiological responses: ΔQTi ~16.5 (vs. 24.5 in sham), and ΔAPD90 ~109 ms. Ca^2+^ transient duration (Ca^2+^T90) also normalized (baseline 338 ± 13 ms to 293 ± 17 ms after ISO, *p* = 0.003). The treatment reduced mutant KCNQ1 mRNA by ~30% and decreased QT dispersion. No significant inflammation or adverse events were observed.
Dotzler et al. (2023) [108]	Congenital Long QT Syndrome Type 1 (LQT1)	Preclinical (transgenic rabbit model)	AAV9	Suppression-and-replacement of KCNQ1 using AAV-shRNA + shRNA-immune KCNQ1 cDNA	Combined suppression of endogenous mutant KCNQ1 mRNA and replacement with shRNA-resistant KCNQ1 cDNA via AAV9 resulted in shortened QT interval and normalized electrophysiological parameters. QTc shortened by ~13% post-treatment (from 457 ± 21 ms to 397 ± 17 ms, *p* < 0.01). Action potential duration at 90% repolarization (APD90) in isolated cardiomyocytes reduced from 499 ± 19 ms (sham) to 399 ± 15 ms (treated), approximating wild-type values. β-adrenergic response (isoproterenol challenge) restored: ΔAPD90 increased by 86 ± 10 ms in treated vs. 39 ± 8 ms in sham (*p* < 0.01). Ventricular arrhythmia inducibility was significantly reduced. No adverse inflammation or off-target effects observed.
Bains et al. (2023) [31]	Congenital Long QT Syndrome Type 2 (LQT2)	Preclinical (KCNH2-mutant rabbit model)	AAV9	Suppression-and-replacement of KCNH2 via shRNA + shRNA-immune KCNH2 cDNA	The AAV9 vector delivered a dual approach: shRNA suppressed endogenous mutant KCNH2, while shRNA-immune KCNH2 cDNA restored normal function. QTc duration was significantly reduced from 470 ± 11 ms in sham to 414 ± 12 ms in treated animals (*p* < 0.01). Cardiomyocyte APD90 decreased from 519 ± 19 ms (sham) to 424 ± 13 ms (treated), approaching WT values (400 ± 9 ms). β-adrenergic stimulation (isoproterenol) led to preserved physiological APD90 shortening in treated animals (ΔAPD90 = −90 ms) vs. minimal change in sham (−36 ms). Early afterdepolarizations (EADs) were significantly reduced, and inducibility of Torsades de Pointes was suppressed.
	Congenital Short QT Syndrome Type 1 (SQT1)	Preclinical (transgenic rabbit model)	AAV9	Gain-of-function KCNH2 suppression via shRNA (no replacement)	In SQT1 rabbits (gain-of-function KCNH2 N588K mutation), shRNA targeting mutant KCNH2 significantly prolonged QTc from 283 ± 8 ms to 333 ± 9 ms (*p* < 0.01), approaching wild-type values (342 ± 7 ms). Cardiomyocyte APD90 increased from 267 ± 11 ms to 316 ± 9 ms. Ventricular effective refractory period (VERP) improved from 142 ± 8 ms to 187 ± 11 ms (*p* < 0.05). Arrhythmia inducibility was significantly reduced (Torsades observed in 5/7 sham vs. 1/8 treated; *p* = 0.03). No off-target effects or myocardial inflammation were detected.
Qi et al. (2024) [87]	Congenital Long QT Syndrome Type 3 (LQT3)	Preclinical (Scn5a-M1875T knock-in mouse model)	AAV9	In vivo base editing using ABE8e-SpRY to correct SCN5A-M1875T	AAV9-encoded adenine base editor (ABE8e-SpRY) was delivered systemically at P10. In vivo base editing efficiency reached 54.1% in cardiac tissue. Corrected mice exhibited significantly shorter QTc intervals (from 57.3 ± 1.3 ms to 47.6 ± 1.2 ms; *p* < 0.0001), normalized action potential duration (APD90 from 58.9 ± 2.1 ms to 45.7 ± 1.4 ms), and restored sodium channel function (reduction in late sodium current by 66%). Arrhythmia burden decreased significantly (6/10 untreated vs. 1/11 treated mice with inducible VT; *p* < 0.01). No detectable off-target edits or adverse effects were reported.
Bradford et al. (2023) [113]	Arrhythmogenic right ventricular cardiomyopathy (ARVC)	Preclinical (mouse)	AAV9	AAV-mediated PKP2 replacement	In PKP2 IVS10-1G>C knock-in mice, early AAV-PKP2 delivery at postnatal day 2 restored PKP2 expression, desmosomal protein levels (DSP, DSG2, JUP), and gap junction protein CX43, fully preventing ARVC phenotype and ensuring 100% survival up to 6 months. Late-stage administration at 4 weeks also significantly improved cardiac function, reduced fibrosis, normalized ECG (QRS duration), eliminated PVCs (0% vs. 60% in controls), and improved survival to 100% at 20 weeks vs. 20% in GFP controls. Rescue occurred even at advanced disease stages.
van Opbergen et al. (2024) [114]	Arrhythmogenic right ventricular cardiomyopathy (ARVC)	Preclinical (mouse model)	AAV9	AAV-mediated PKP2a gene transfer	AAV9-mediated delivery of *Plakophilin-2a (PKP2a)* in PKP2-deficient mice arrested disease progression. Early treatment (2 days after birth) restored intercalated disc protein expression (Cx43, N-cadherin, desmoplakin) and prevented right ventricular dilation and dysfunction. Late-stage therapy (initiated at 4 weeks) reduced arrhythmias, restored conduction velocity, and improved survival (100% vs. 20% in controls at 20 weeks).

Abbreviations: AAV, adeno-associated virus; AAV9, adeno-associated virus serotype 9; ABE8e-SpRY, adenine base editor version 8e with SpRY Cas9 variant; APD, action potential duration; APD90, action potential duration at 90% repolarization; APD100, action potential duration at 100% repolarization; ARVC, arrhythmogenic right ventricular cardiomyopathy; BrS, Brugada syndrome; CaMKII, calcium/calmodulin-dependent protein kinase II; CCD, cardiac conduction disease; CPVT1, catecholaminergic polymorphic ventricular tachycardia type 1; Cx43, connexin 43; DSp, desmoplakin; DSG2, desmoglein-2; EAD, early afterdepolarization; EF, ejection fraction; FS, fractional shortening; GFP, green fluorescent protein; hiPSC, human induced pluripotent stem cell; hiCM, human induced pluripotent stem cell-derived cardiomyocyte; I_Na_, sodium current; ISO, isoproterenol; JUP, junction plakoglobin; jSR, junctional sarcoplasmic reticulum; KO, knockout; KCNH2, potassium voltage-gated channel subfamily H member 2; KCNQ1, potassium voltage-gated channel subfamily Q member 1; LQT1, long QT syndrome type 1; LQT2, long QT syndrome type 2; LQT3, long QT syndrome type 3; MOG1, monomeric GTP-binding protein 1; PVC, premature ventricular contraction; RyR2, ryanodine receptor 2; SaCas9, Staphylococcus aureus CRISPR-associated protein 9; SCN5A, sodium voltage-gated channel alpha subunit 5; shRNA, short hairpin RNA; siRNA, small interfering RNA; SND, sinus node dysfunction; SupRep, suppression-and-replacement; SV, stroke volume; TrD, triadin; VG, viral genome; VERP, ventricular effective refractory period; VT, ventricular tachycardia.

The success of SupRep therapy hinges on achieving a finely tuned balance between the suppressive and replacement components [115]. Disproportionate suppression in the absence of adequate replacement may aggravate the LQTS phenotype, whereas excessive replacement with insufficient suppression risks inducing a short QT phenotype [115]. To address this challenge, investigators have employed strategies such as synchronized expression of both components under cardiac-specific promoters, iterative dose-escalation studies in animal models, and in silico modeling to optimize dosing precision [115]. Additionally, rigorous post-treatment monitoring using electrocardiography and molecular biomarkers is essential to ensure therapeutic efficacy and minimize adverse effects. Collectively, these preclinical findings—particularly the successful in vivo phenotypic rescue in a large-animal model—underscore the translational promise of SupRep gene therapy. Beyond LQTS, this modular and scalable approach holds potential for adaptation to a broad spectrum of monogenic cardiovascular disorders characterized by dominant-negative pathophysiology [115].

Pathogenic variants in SCN5A, the gene encoding the cardiac sodium channel Na_v_1.5, are implicated in two distinct electrophysiological disorders: Brugada syndrome, associated with loss-of-function mutations, and long QT syndrome type 3 (LQT3), caused by gain-of-function variants. While SCN5A remains an attractive therapeutic target, its large coding sequence (~6048 base pairs) exceeds the packaging capacity of adeno-associated virus (AAV) vectors, necessitating alternative approaches.

In Brugada syndrome, AAV9-mediated overexpression of MOG1 (Ran guanine nucleotide release factor), a critical modulator of Na_v_1.5 trafficking, was employed in a knock-in mouse model harboring the SCN5A^G1746R^ mutation [112]. This strategy enhanced the cell surface localization of Na_v_1.5 channels and reversed the arrhythmic phenotype, demonstrating the feasibility of pathway-targeted gene therapy as an alternative to direct SCN5A replacement in the context of vector size constraints [112].

For LQT3, a novel gene-editing approach utilizing adenine base editing was applied to a newly established mouse model carrying the SCN5A^T1307M^ mutation [87]. The adenine base editor was split into two components and delivered via a dual AAV9 vector system through a single intraperitoneal injection [87]. Remarkably, while complete QT interval normalization required approximately 60% transcript-editing efficiency, even modest editing levels (~20%) were sufficient to prevent life-threatening arrhythmias [87]. This observation may be explained by the source–sink principle in cardiac electrophysiology, whereby electrotonic coupling through gap junctions enables non-arrhythmic cardiomyocytes to buffer and dissipate aberrant electrical activity from neighboring cells, thereby preventing the propagation of malignant rhythms at the tissue level [116].

Although arrhythmogenic right ventricular cardiomyopathy (ARVC) is not classified as a primary channelopathy, it shares significant overlap in its arrhythmogenic potential and clinical presentation with inherited electrical disorders. The most prevalent form of desmosomal ARVC results from loss-of-function mutations in PKP2, which encodes plakophilin-2—a critical component of the cardiac desmosome. These mutations compromise structural integrity and intercellular adhesion, ultimately promoting myocardial fibrosis, biventricular dysfunction, and life-threatening arrhythmias. Preclinical studies have provided compelling evidence for the efficacy of gene replacement therapy (GRT) targeting PKP2 [113,117]. In two independent murine models of ARVC, systemic delivery of PKP2 via viral vectors restored plakophilin-2 expression, resulting in significant improvements in left ventricular ejection fraction, attenuation of fibrotic remodeling, suppression of arrhythmic burden, and enhanced overall survival—even when therapy was initiated after the onset of overt disease phenotypes [117].

Building upon these foundational studies, multiple PKP2-targeted GRT candidates have progressed into early-phase clinical trials. These include LX2020 (Lexeo Therapeutics) [118], RP-A601 (Rocket Pharmaceuticals) [119], and TN-401 (Tenaya Therapeutics) [120], all of which represent first-in-class efforts to translate desmosomal gene therapy into viable treatment options for patients with ARVC.

### 2.3. Gene Therapy Approaches for Biological Pacemaker Development

While electronic pacemakers remain the cornerstone of therapy for bradyarrhythmias and conduction system disease, they are not without limitations [121]. Complications such as lead dislodgement, generator failure, device-related infections, lack of physiologic autonomic responsiveness, and vulnerability to electromagnetic interference underscore the need for alternative, biologically integrated pacing solutions. In this context, biological pacemakers have emerged as a promising therapeutic paradigm aimed at restoring intrinsic cardiac automaticity [122,123].

Biological pacemakers are designed to induce or augment automatic impulse generation by creating a functional ectopic pacemaker site that mimics sinoatrial node activity [122]. Several gene therapy-based strategies have been investigated to this end, including receptor-based modulation, ion channel re-engineering, and gene–cell hybrid approaches [123].

Among these, somatic reprogramming has garnered considerable attention for its ability to convert non-pacemaker cardiomyocytes into pacemaker-like cells through targeted transcriptional manipulation [59]. A particularly compelling strategy involves overexpression of the T-box transcription factor TBX18, a key developmental regulator of sinoatrial node identity [59]. Experimental studies have demonstrated that TBX18 gene delivery can reprogram ventricular cardiomyocytes into cells exhibiting pacemaker-like electrophysiological properties both in vitro and in vivo [60]. In a porcine model of complete atrioventricular block, AAV-mediated TBX18 transfer successfully induced sustained biological pacemaker activity with favorable safety and functional profiles [60].

Another recent large-animal study provided compelling evidence supporting the use of synthetic mRNA for biological pacemaker development [62]. In a porcine model of complete atrioventricular block, chemically modified TBX18 mRNA was delivered via intramyocardial injection to reprogram ventricular cardiomyocytes into pacemaker-like cells [62]. The reprogrammed tissue exhibited key molecular and electrophysiological features of sinoatrial node cells, including spontaneous diastolic depolarization, reduced resting membrane potential, and expression of nodal genes such as HCN4 and SHOX2 [62]. Importantly, this mRNA-based therapy induced autonomically responsive escape rhythms that persisted for up to two weeks without requiring electronic pacing support [62]. The biological pacing demonstrated appropriate rate modulation following β-adrenergic stimulation and vagal blockade, highlighting functional integration with the autonomic nervous system. Moreover, no adverse immune responses or ventricular arrhythmias were observed, underscoring the safety advantages of non-viral delivery platforms [62].

Modulation of specific ion currents represents a mechanistically targeted approach to the generation of biological pacemakers. One such strategy involves suppressing the inward rectifier potassium current (I_K1_), which plays a pivotal role in stabilizing the resting membrane potential of ventricular cardiomyocytes. This has been achieved through the use of a dominant-negative mutant of the KCNJ2-encoded Kir2.1 channel (Kir2.1AAA), effectively reducing I_K1_ density and thereby inducing spontaneous depolarization [124]. While this method successfully initiates automaticity in otherwise quiescent myocytes, it carries inherent risks—including excessive action potential prolongation and augmented dispersion of repolarization—which may predispose to proarrhythmic events [125].

An alternative and more physiologically aligned approach involves augmenting the hyperpolarization-activated, cyclic nucleotide-gated (HCN) channel-mediated current (I_K1_), which underlies diastolic depolarization in native pacemaker cells [126]. Early preclinical studies using HCN2 overexpression in canine models demonstrated the induction of stable pacemaker activity with autonomic responsiveness; however, pacing rates achieved were below physiologic targets [126]. To address these limitations, researchers engineered mutant HCN constructs with enhanced sensitivity to catecholamines, thereby emulating the dynamic autonomic modulation intrinsic to sinoatrial node function [127]. Moreover, dual gene delivery strategies—combining HCN channel overexpression with co-expression of hyperpolarizing elements—have been investigated to further refine the electrophysiological profile and improve pacing efficacy [128].

Beyond gene- and ion channel-based approaches, alternative strategies are being actively explored to engineer biological pacemakers with enhanced physiological integration and functional precision. Tissue engineering techniques have enabled the generation of sinoatrial node–like pacemaker cells from human induced pluripotent stem cells (hiPSCs) through transgene-independent modulation of developmental signaling pathways [129]. These bioengineered cells, once transplanted into rat myocardium, have demonstrated the capacity to integrate structurally and functionally, recapitulating key features of the native sinoatrial node [129]. Moreover, optogenetic technologies offer a novel, non-invasive modality for controlling cardiac excitability [130]. By harnessing light-sensitive ion channels such as channelrhodopsins, optogenetics allows for the precise temporal and spatial modulation of cardiomyocyte activity, presenting an innovative avenue for rhythm control. Preliminary studies have shown the feasibility of this technique in modulating pacemaker function with high fidelity and responsiveness [131,132,133].

Although these approaches are still in preclinical stages, accumulating evidence supports their potential to complement or replace traditional pacing systems [62,134,135,136,137]. Nonetheless, rigorous investigation into their long-term safety, immunogenicity, integration efficiency, and electrophysiological stability in large-animal models is essential before consideration for clinical translation [122].

### 2.4. Routes of Administration in Cardiac Gene Therapy

The route of vector delivery greatly affects transduction efficiency, heart coverage, tissue specificity, and the overall success of cardiac gene therapy [138,139,140]. While the vector type determines its tropism and how long it expresses, the delivery method influences how much gene transfer occurs in the heart and how evenly it distributes, impacting both effectiveness and safety [141].

Intravenous (IV) injection is the easiest and least invasive method, allowing for systemic vector delivery [142]. However, IV delivery often leads to significant off-target distribution, especially in the liver with AAV vectors. This results in less effective gene transfer to the heart and a higher risk of systemic toxicity. This issue is particularly significant when large doses of the vector are necessary for treatment.

Intracoronary infusion is typically performed using a catheter [143,144]. This method directly delivers the vector into the coronary blood vessels, which improves uptake in the heart. It also allows for wider distribution throughout the heart muscle, especially when used alongside medications like vasodilators or agents that cause temporary ischemia [143]. Still, its effectiveness can decrease in patients with blocked coronary arteries or poor blood flow in small vessels. The procedure also carries risks typical of invasive catheterization.

Intramyocardial injection, which can be performed through the outer or inner heart surface, allows for targeted delivery to specific heart regions [145]. This method is often used in research and during certain heart surgeries. While it provides focused gene expression with minimal systemic exposure, its invasive nature and limited spread make it less useful for widespread heart diseases. Additionally, uneven distribution of the gene can lead to localized electrical disturbances or inflammation, raising concerns about arrhythmias.

Ultimately, the best route for administration depends on various factors, including the vector type, the target area in the heart, the disease involved, and the clinical context [146]. Upcoming strategies might use image-guided or electromechanically mapped delivery, magnetically guided nanoparticles, or advanced catheter systems to improve precision and reduce the risks of procedures. Combining considerations of delivery routes with vector design will be crucial for the success of cardiac gene therapy.

## 3. Limitations and Future Directions

Although gene therapy has emerged as a transformative approach in the treatment of cardiac arrhythmias, several critical limitations hinder its full clinical realization. A foremost challenge remains the lack of precise, cardiac-specific delivery systems. Current vectors, including adeno-associated viruses (AAVs), exhibit suboptimal myocardial tropism and often accumulate in off-target organs, particularly the liver. This reduces therapeutic efficiency and increases the risk of systemic toxicity. While antibody–oligonucleotide conjugates and other targeted delivery platforms have shown promise in non-cardiac diseases, their application in cardiac gene therapy remains limited and warrants further investigation [147,148,149,150].

In addition to these delivery-related constraints, concerns regarding off-target effects and long-term safety remain central to the clinical translation of cardiac gene therapy. Despite progressive improvements in vector design, unintentional transduction of non-cardiac tissues—most notably hepatocytes and skeletal muscle—can trigger immune responses or ectopic gene expression, with potentially deleterious consequences. Although AAV vectors are largely episomal, rare cases of genomic integration have been reported, posing a theoretical risk of insertional mutagenesis. Equally important is the uncertain long-term efficacy of gene therapies in post-mitotic cardiac tissues, where vector persistence, epigenetic silencing, or immune-mediated clearance may compromise durability. These issues underscore the need for extended preclinical follow-up and rigorous post-marketing surveillance to evaluate delayed adverse events and inform regulatory standards.

Another significant barrier is the restricted packaging capacity of AAV vectors, which precludes the delivery of large or complex genes frequently implicated in inherited arrhythmia syndromes (e.g., RYR2 and SCN5A). Approaches such as dual-vector systems, trans-splicing, and the use of mini-gene constructs have been proposed, yet these strategies suffer from inconsistent efficiency and unpredictable expression dynamics [151]. Furthermore, host immune responses to viral vectors, whether pre-existing or treatment-induced, can limit therapeutic efficacy and prevent redosing. Emerging strategies—including transient immunosuppression, capsid re-engineering, and extracellular vesicle shielding—may mitigate these effects, but remain in early developmental stages.

The translational gap between preclinical models and human electrophysiology also poses a significant challenge [152,153,154]. While murine models are indispensable for early-stage screening, their divergent heart rates and ion channel profiles limit clinical relevance. Intermediate species (e.g., rabbits and guinea pigs) offer closer physiological parallels but are genetically less tractable. Large-animal models, particularly pigs, provide a more faithful representation of human cardiac structure and conduction properties; however, their use is constrained by high cost and logistical complexity. To ensure robust preclinical validation, future studies must prioritize systematic testing in large-animal models and, eventually, non-human primates.

Looking forward, several avenues offer potential to accelerate the field. The development of next-generation vectors with enhanced cardiac specificity and immune evasiveness, coupled with non-viral delivery modalities such as mRNA-based approaches, could broaden the applicability and safety of cardiac gene therapies. Additionally, advances in computational modeling, patient-derived cardiomyocytes, and digital twin platforms may enable personalized prediction of arrhythmia susceptibility and therapeutic response [155,156]. Finally, robust strategies for long-term safety monitoring, precise dosing control, and regulatory oversight will be essential to guide the responsible translation of gene therapy technologies from experimental models to clinical practice [157,158].

## 4. Conclusions

Genetic modulation strategies are rapidly emerging as a promising avenue for the treatment of both inherited and acquired cardiac arrhythmias. By targeting the underlying molecular and electrophysiological abnormalities—rather than only addressing the symptoms—gene-based therapies offer the potential for more durable and disease-modifying effects. Advances in gene silencing, replacement, and editing technologies have enabled preclinical success across a range of arrhythmia syndromes, including long QT syndrome, catecholaminergic polymorphic ventricular tachycardia, and atrial fibrillation. Despite these encouraging developments, several challenges remain. Limitations in vector specificity, immune responses, delivery efficiency, and the complexity of arrhythmogenic mechanisms continue to constrain clinical translation. Additionally, differences between animal models and human cardiac physiology necessitate further validation in large-animal systems and well-designed clinical trials.

Continued progress will depend on the integration of advanced delivery systems, non-viral platforms, and personalized modeling approaches, including patient-derived cardiomyocytes and in silico simulations. As these technologies evolve, gene therapy may gradually assume a more central role in the management of arrhythmias, complementing existing strategies and potentially offering durable, mechanism-based interventions for selected patient populations.

## Figures and Tables

**Table 1:** Key Studies Investigating Gene Therapy Strategies for Acquired Cardiac Arrhythmias.

Author (Year)	Type of Arrhythmia	Type of Study	Vector	Form of Gene Therapy	Main Findings
Amit et al. (2010) [51]	Atrial fibrillation	Preclinical (porcine model)	Adenovirus (AdG628S)	Dominant-negative gene transfer (KCNH2-G628S)	Atrial painting with AdG628S prolonged atrial MAPD_90_ significantly at day 7 (right atrium: median increase ~80 ms vs. control, *p* < 0.01), with all treated animals maintaining sinus rhythm (SR) from days 4 to 10. By day 21, loss of transgene expression led to loss of efficacy. Relative risk of AF was 0.44 (95% CI 0.33–0.59; *p* < 0.01) in treated animals. KCNH2 protein expression was 79% higher in the treated group at day 7. No ventricular proarrhythmia or electrophysiological changes observed.
Soucek et al. (2012) [50]	Atrial fibrillation	Preclinical (porcine model)	Adenovirus (AdCERG-G627S)	Dominant-negative gene transfer (CERG-G627S)	AdCERG-G627S significantly delayed or prevented persistent AF in pigs (mean time to persistent AF: 12 ± 2.1 days vs. 6.2 ± 1.3 days in controls; *p* < 0.001). Atrial ERP and MAPD90 were significantly prolonged (ERP: 221.5 ± 4.7 ms vs. 197.0 ± 4.7 ms; *p* = 0.006). Left ventricular function was preserved (LVEF: 62.1% ± 4.0% vs. 30.3% ± 9.1%; *p* < 0.001). No ventricular or extracardiac expression observed, supporting atrial-specific safety of the therapy.
Bikou et al. (2011) [55]	Atrial fibrillation	Preclinical (porcine model)	Adenovirus (AdKir2.1AAA)	Dominant-negative gene transfer (Kir2.1AAA)	AdKir2.1AAA selectively suppressed I_K1_ current, leading to significant prolongation of atrial ERP (from 131 ± 5 ms to 174 ± 6 ms; *p* < 0.001). AF inducibility was markedly reduced (AF episodes: 0.4 ± 0.2 vs. 2.7 ± 0.3 in controls; *p* < 0.001). No significant ventricular ERP prolongation or proarrhythmic effects observed. Protein expression confirmed selective atrial Kir2.1AAA expression without extracardiac transduction.
Igarashi et al. (2011) [54]	Atrial fibrillation	Preclinical (porcine model)	Adenovirus (AdCx40, AdCx43)	Overexpression (connexin 40 and 43)	AdCx40 and AdCx43 gene transfer preserved atrial conduction velocity (41.2 ± 3.2 cm/s vs. 26.5 ± 3.5 cm/s in controls; *p* < 0.001) and prevented AF induction at 7 days. AF inducibility was reduced by >80% (induction rate: 12.5% vs. 66.7% in controls; *p* < 0.01). Connexin expression was successfully upregulated and localized at intercalated disks. No adverse effects observed in ventricular conduction or function.
Kunamalla et al. (2016) [56]	Atrial fibrillation	Preclinical (canine model)	Lentivirus (LVdnTGFβRII)	Inhibition of TGF-β signaling (dominant-negative receptor)	Posterior LA gene transfer of LVdnTGFβRII significantly reduced atrial fibrosis (collagen volume fraction: 13.9% ± 2.4% vs. 27.1% ± 1.7%; *p* < 0.05) and preserved conduction velocity. AF duration was significantly shorter (mean 1.3 ± 0.4 s vs. 5.6 ± 1.1 s; *p* < 0.05), with fewer sustained AF episodes. No off-target effects in ventricular myocardium or extracardiac organs detected. The anti-fibrotic strategy preserved posterior wall structure and conduction.
Trappe et al. (2013) [57]	Ventricular tachycardia (post-infarction)	Preclinical (porcine model)	Adenovirus (AdCx43)	Overexpression (connexin 43)	AdCx43 gene transfer into the infarct border zone significantly increased Cx43 protein expression (1.8 fold; *p* < 0.01) and improved electrical coupling. Ventricular conduction velocity was significantly faster (25.8 ± 3.4 vs. 16.2 ± 2.1 cm/s in controls; *p* < 0.01), and inducibility of sustained VT was reduced by 50%. Reduced arrhythmia burden was associated with homogeneous Cx43 distribution and improved connexin-mediated intercellular coupling. No increase in proarrhythmia or off-target expression observed.
Aistrup et al. (2009) [58]	Atrial fibrillation	Preclinical in vivo (canine)	Adenoviral vector (AdGαi2ctp)	Gαi2 C-terminal peptide overexpression (dominant-negative G-protein inhibition)	Epicardial atrial gene transfer of AdGαi2ctp significantly reduced AF duration from 13.0 ± 4.2 s (control) to 0.8 ± 0.5 s at 3–5 days post-transduction (*p* < 0.001). AF inducibility was also markedly decreased. The intervention selectively attenuated I_KAch activation and prolonged action potential duration during vagal stimulation, without affecting sympathetic signaling. Histological assessment confirmed targeted expression with minimal inflammatory response.
Kapoor et al. (2013) [59]	Bradyarrhythmia (Sinoatrial node dysfunction)	In vitro and in vivo (animal model—guinea pig)	Adenoviral vector	Ectopic overexpression of Tbx18 transcription factor	Tbx18 reprogrammed ventricular myocytes into sinoatrial node-like pacemaker cells (iSAN) with spontaneous beating. In vitro, 73.8% of Tbx18-NRVMs beat spontaneously vs. 28.8% in controls (*p* < 0.05). Action potential frequency was 95 ± 23 bpm vs. 46 ± 10 bpm in controls. In vivo, 5/7 Tbx18-injected guinea pigs showed ectopic ventricular rhythm; beating rate ~154 bpm vs. ~120 bpm in controls. Converted cells had reduced IK1 (78% decrease), depolarized MDP (−47 ± 10 mV vs. −73 ± 6 mV), increased HCN4 (3.8× more HCN4+ cells), and durable pacemaker phenotype up to 8 weeks despite loss of Tbx18 transcript.
Hu et al. (2014) [60]	Ventricular tachyarrhythmia post-myocardial infarction	In vivo (canine model of MI)	Adenoviral vector (AdCx43)	Overexpression of connexin 43 (Cx43) in the infarct border zone	Cx43 gene transfer restored gap junction expression and improved conduction velocity post-MI. Inducibility of sustained VT was reduced to 10% in treated animals vs. 70% in controls (*p* < 0.01). Conduction velocity increased from 13.1 ± 1.6 cm/s (control) to 21.3 ± 1.7 cm/s (treated, *p* < 0.001). Cx43 protein expression doubled, and immunostaining confirmed localization to intercalated disks. Gene expression peaked at 72 h and declined thereafter, but antiarrhythmic effects were sustained for ≥1 week.
Lau et al. (2009) [61]	Post-infarction ventricular arrhythmia	In vivo (canine model of myocardial infarction)	Adenoviral vector (AdSkM1-GFP)	Overexpression of skeletal muscle sodium channel SkM1 in the epicardial border zone	SkM1 gene transfer restored fast sodium current in the infarct border zone, improving conduction and reducing arrhythmogenicity. Treated hearts had faster epicardial activation (median CV 47.2 cm/s vs. 27.3 cm/s in controls, *p* < 0.001) and preserved conduction delay. Transduced cells showed normal excitability at depolarized resting membrane potentials, unlike native cardiac Na+ channels. Histology confirmed successful transgene expression localized to the border zone myocytes. No increase in arrhythmia susceptibility observed post-intervention.
Wolfson et al. (2024) [62]	Complete AV block (CAVB)	Preclinical in vivo (rat and pig)	Synthetic mRNA (naked, unformulated)	TBX18 mRNA (non-viral gene therapy)	Myocardial injection of TBX18 mRNA in rats and pigs induced de novo biological pacing. In rats, heart rates were significantly higher vs. controls over 14 days (mean HR ~343 bpm vs. ~130 bpm, *p* < 0.01). In pigs, pacing was rate-adaptive and correlated with physical activity (Pearson’s R = 0.67 vs. 0.21 in controls). TBX18 mRNA achieved focal expression, low inflammation, and significantly reduced backup pacemaker dependency over 28 days.
Anttila et al. (2023) [63]	Ischemic cardiomyopathy	Randomized controlled trial (phase 2a)	Naked mRNA (AZD8601, VEGF-A mRNA)	mRNA-mediated protein expression (VEGF-A)	In the EPICCURE trial (NCT03370887), 30 epicardial injections of AZD8601 (3 mg total) during CABG were safe, with no arrhythmias, infections, or immune reactions observed. Exploratory outcomes suggested improved LVEF, NT-proBNP reduction, and better KCCQ scores over 6 months in treated vs. placebo groups. This study provides first-in-human evidence of the safety and feasibility of direct myocardial injection of naked VEGF-A mRNA without lipid encapsulation.

Abbreviations: AF, atrial fibrillation; VT, ventricular tachycardia; ERP, effective refractory period; MAPD90, action potential duration at 90% repolarization; LVEF, left ventricular ejection fraction; Cx40, connexin 40; Cx43, connexin 43; SK2 and SK3, small-conductance calcium-activated potassium channel subtypes 2 and 3; I_K1_, inward rectifier potassium current; I_Ks_, slowly activating delayed rectifier potassium current; I_SK_, small-conductance calcium-activated potassium current; MI, myocardial infarction; LNP, lipid nanoparticle; mRNA, messenger RNA; TGF-β, transforming growth factor beta; GJA1, gene encoding connexin 43; dn, dominant negative; Ad, adenovirus vector; LV, lentivirus vector; LNP-GJA1-mRNA, lipid nanoparticle–encapsulated GJA1 mRNA used to induce transient Cx43 overexpression.

## Data Availability

All data generated in this research are included in this article.
